# High-Dose Ascorbate in Combination with Anti-PD1 Checkpoint Inhibition as Treatment Option for Malignant Melanoma

**DOI:** 10.3390/cells12020254

**Published:** 2023-01-07

**Authors:** Markus Burkard, Heike Niessner, Christian Leischner, Alban Piotrowsky, Olga Renner, Luigi Marongiu, Ulrich M. Lauer, Christian Busch, Tobias Sinnberg, Sascha Venturelli

**Affiliations:** 1 Department of Nutritional Biochemistry, Institute of Nutritional Sciences, University of Hohenheim, Garbenstraße 30, 70599 Stuttgart, Germany; 2 Division of Dermatooncology, Department of Dermatology, University of Tuebingen, Liebermeisterstraße 25, 72076 Tuebingen, Germany; 3 Cluster of Excellence iFIT (EXC 2180) “Image-Guided and Functionally Instructed Tumor Therapies”, 72076 Tuebingen, Germany; 4 Department of Internal Medicine VIII, University Hospital Tuebingen, Otfried-Mueller-Straße 10, 72076 Tuebingen, Germany; 5 Dermatologie zum Delfin, Stadthausstraße 12, 8400 Winterthur, Switzerland; 6 Department of Dermatology, Venereology and Allergology, Charité-Universitätsmedizin Berlin, Charitéplatz 1, 10117 Berlin, Germany; 7 Department of Vegetative and Clinical Physiology, Institute of Physiology, University of Tuebingen, Wilhelmstraße 56, 72074 Tuebingen, Germany

**Keywords:** malignant melanoma, cancer, ascorbate, vitamin C, immunotherapy, checkpoint blockade, B16F10 melanoma cells

## Abstract

Ascorbate acts as a prooxidant when administered parenterally at high supraphysiological doses, which results in the generation of hydrogen peroxide in dependence on oxygen. Most cancer cells are susceptible to the emerging reactive oxygen species (ROS). Accordingly, we evaluated high-dose ascorbate for the treatment of the B16F10 melanoma model. To investigate the effects of ascorbate on the B16F10 cell line in vitro, viability, cellular impedance, and ROS production were analyzed. In vivo, C57BL/6N^Crl^ mice were subcutaneously injected into the right flank with B16F10 cells and tumor-bearing mice were treated intraperitoneally with ascorbate (3 g/kg bodyweight), immunotherapy (anti-programmed cell death protein 1 (PD1) antibody J43; 2 mg/kg bodyweight), or both treatments combined. The efficacy and toxicity were analyzed by measuring the respective tumor sizes and mouse weights accompanied by histological analysis of the protein levels of proliferating cell nuclear antigen (Pcna), glucose transporter 1 (Glut-1), and CD3. Treatment of B16F10 melanoma-carrying mice with high-dose ascorbate yielded plasma levels in the pharmacologically effective range, and ascorbate showed efficacy as a monotherapy and when combined with PD1 inhibition. Our data suggest the applicability of ascorbate as an additional therapeutic agent that can be safely combined with immunotherapy and has the potential to potentiate anti-PD1-based immune checkpoint blockades.

## 1. Introduction

The lifetime risk to develop melanoma is about 2.4% for the Caucasian population in the United States, and malignant melanoma causes most skin cancer-associated deaths due to early metastasis [1,2]. Its incidence is steadily increasing worldwide [2]. Until 2010, chemotherapeutics had largely failed to prolong overall survival (OS) in metastatic melanoma patients, and significant survival benefits were first achieved with the introduction of selective inhibitors of oncogenic B-Raf kinase (B-rapidly accelerated fibrosarcoma; BRAFi), which is constitutively active in about 50% of melanoma patients (vemurafenib was approved in 2011 and dabrafenib in 2013, both with high initial efficacy but often fast-occurring resistances [3,4,5]), as well as with the introduction of the antibody ipilimumab against cytotoxic T-lymphocyte-associated protein 4 (CTLA-4) in 2011, followed by the approval of PD1 checkpoint inhibitors by the U.S. Food and Drug Administration (FDA) for the treatment of metastatic melanoma in 2014 [6,7,8]. The broad implementation of these antibodies into treatment protocols has largely changed disease management options. As of 2021, the five-year survival rate for localized melanoma was about 98.3% and about 44% for metastatic melanoma [7,9]. For patients with unresectable advanced stages, targeting PD1 provides a significant benefit by blocking immunoinhibitory signaling in T cells and enables melanoma patients to adequately respond to the tumor [10,11]. Treatment with the PD1 antibody nivolumab resulted in sustained tumor regression in several patients [12,13]. Complete or partial response was observed in a third or more of advanced melanoma (stage IIIC or IV) patients, significantly increasing patients’ five-year survival [13,14,15]. However, about 40% of the patients did not respond to anti-PD1-based immune checkpoint blockades [16].

Even though inhibiting PD1 prolongs the OS by activating T-cell-mediated antitumor immune responses [12], a substantial number of patients progress because of primary or acquired resistance [17,18]. Therefore, although novel immune therapies (checkpoint inhibitors) and BRAFi have significantly improved the disease prognosis, there is still an immense need for additional therapeutic approaches. 

Decades ago, ascorbate was suggested to improve the OS of advanced-stage cancer patients [19,20], and initial in vivo studies demonstrated anticancer activity of supraphysiological doses of ascorbate when administered parenterally [21,22,23,24]. Clinically relevant, high-dose ascorbate was also well tolerated, and even decreased unwanted side effects of chemotherapy [21]. Unfortunately, this therapy was no longer pursued because ascorbate was shown not to have significant antitumor effects in several studies [25,26]. A major reason for this lack of efficacy was the oral route of administration in these follow-up studies, which is not suitable for achieving supraphysiological levels of ascorbate (>220 µM) or even pharmacological plasma levels in the millimolar range [27,28,29]. 

In recent years, ascorbate has experienced a kind of renaissance in tumor therapy. Examples for ascorbate efficacy include increased chemosensitivity of ovarian cancer associated with reduced chemotherapeutic toxicity and preferential killing of hepatocellular cancer stem cells involving sodium-dependent vitamin C transporter type 2 (SVCT2; *SLC23A2*), with very few cytotoxic effects on normal cells [30,31]. It was also described that intravenous high-dose ascorbate selectively killed Kirsten rat sarcoma virus (KRAS)-or BRAF-mutated cells from colorectal cancers [32]. Additionally, vitamin C increased the function of immune cells such as natural killer cells, dendritic cells, and macrophages [33,34]. Recent results indicate that high-dose ascorbate treatment overcomes distinct resistance mechanisms against checkpoint inhibitors, and ascorbate enhances tumor recognition by the host immune system, yielding increased infiltration of macrophages and cytotoxic T cells into the tumor compared with anti-PD1 monotherapy, suggesting synergistic antitumoral mechanisms of the combined therapy in a murine lymphoma model and further in colorectal, breast, and pancreatic cancer mouse models [35,36]. Moreover, the combination of high-dose ascorbate and targeted therapy with vemurafenib demonstrated promising effects of the additional use of ascorbate in melanoma-bearing mice [37]. Our own data have also shown that patients with metastatic melanoma have decreased plasma ascorbate levels, suggesting a combination of standard therapies with vitamin C infusions [38].

In this work, we verified the efficacy of ascorbate to kill melanoma cells in vitro and in vivo. We evaluated the anticancer effects of ascorbate either alone or in combination with the anti-mouse PD1 antibody J43 in a syngeneic C57BL/6N^Crl^ melanoma model and elucidated the modifications of the tumor immune microenvironment.

## 2. Materials and Methods

### 2.1. Melanoma Cell Line

Murine melanoma cells B16F10 were obtained from the American Type Culture Collection (ATCC, Manassas, VA, USA). Cells were periodically examined for mycoplasma contamination with Venor GeM Classic Mycoplasma Detection Kit (Minerva Biolabs, Berlin, Germany).

### 2.2. Treatments

Antibody J43 (hamster anti-mouse PD1 antibody) was purchased from BIO X CELL, Lebanon, USA, and ascorbate (Pascorbin^®^) from Pascoe pharmazeutische Praeparate GmbH, Giessen, Germany.

### 2.3. 4-Methylumbelliferyl Heptanoate Viability Assay

The vitality of the B16F10 cell line was investigated with the 4-methylumbelliferyl heptanoate (MUH) assay (Sigma-Aldrich, Taufkirchen, Germany). Therefore, 3 × 10^4^ cells/well were seeded into 24-well plates, and after 24 h, B16F10 cells were treated in duplicates for 24 h with ascorbate concentrations of 0–5 mM. Quantitative cell decline as positive control was induced by killing cells with 1% (*v*/*v*) Triton X100 (Roth, Karlsruhe, Germany). Prior to analysis, B16F10 cells were washed with PBS and then incubated for 1 h with 100 μg/mL MUH in phosphate-buffered saline (PBS; stock solution 10 mg/mL diluted 1:100) at 37 °C, 5% CO_2_. Hydrolysis of MUH by intracellular esterases and lipases of living cells results in the formation of the strongly fluorescent 4-methylumbelliferone (λ_ex_ 355 nm, λ_em_ 460 nm) and was detected with the Synergy H1 fluorescence microplate reader (BioTek, Bad Friedrichshall, Germany). Fluorescence intensity was proportional to the count of living cells after treatment, yielding the relative number of vital cells (% control).

### 2.4. Sulforhodamine B Cytotoxicity Assay

B16F10 cells (3 × 10^4^ cells/well) were placed into 24-well plates (500 µL medium/well) and incubated for 24 h; 37 °C, 5% CO_2_. After medium change, treatment was performed in duplicates with 0–5 mM ascorbate for 24 h and growth inhibition was evaluated. Quantitative cell death as positive control was induced by adding 1% (*v*/*v*) Triton X100 (Roth) prior to fixing the cells. Then, medium was removed and the wells were washed with cold PBS and fixed with 10% (*v*/*v*) trichloroacetic acid (TCA) for 30 min at 4 °C. After washing the cells with tap water and drying them at 40 °C, protein staining was performed for 10 min with sulforhodamine B reagent (SRB; 0.4% (*w*/*v*) in 1% (*v*/*v*) acetic acid; CAS 3520-42-1, Sigma-Aldrich). Following washing with tap water and 1% (*v*/*v*) acetic acid to remove unbound dye, wells were dried at 40 °C. The protein-bound dye was dissolved with 10 mM Tris base (pH 10.5) for 10 min and absorbance was measured after transferring triplicates of 80 µL/well into a 96-well plate with the SynergyH1 multiplate reader (BioTek; wavelength 550 nm, reference wavelength 620 nm). Data represent the mean of optical density values (% control) as a surrogate parameter for relative cell density. 

### 2.5. Real-Time Proliferation Assay

The xCELLigence^®^ SP system (Agilent Technologies Inc., Waldbronn, Germany) was used to evaluate the cellular impedance as a surrogate parameter for cell proliferation over time. B16F10 cells (4 × 10^3^ cells/well) were seeded in 100 µL medium into a 96-well plate equipped with gold microelectrodes (E-Plate 96, OMNI Life Science GmbH & Co. KG, Bremen, Germany). Prior to this, 90 µL/well of medium had to be provided to measure the zero value per well. Ascorbate treatment was performed in quadruplicates after 24 h in 20 µL/well to reach final concentrations of 0–5 mM in a final volume of 210 µL/well. Quantitative cell decline as positive control was induced by adding 1% (*v*/*v*) Triton X100 (Roth). Measurements of electrical impedance were recorded in 30 min intervals over a period of 72 h. Each experiment was conducted three times. Finally, normalized cell index values (cell index = 1 at 24 h − immediately before treatment) were calculated with the Real-Time Cell Analyzer (RTCA) Software Pro 2.3.2 (Agilent Technologies Inc.).

### 2.6. DCFH-DA-Assay for ROS Detection

B16F10 cells were seeded at a density of 3 × 10^4^ cells per well in 24-well plates in duplicates and then incubated overnight at 37 °C. After 24 h, cells were treated with 0–5 mM ascorbate for different time periods. Three h before the end of treatment, 50 µL of medium were removed from the positive control wells and replaced with 50 µL 10 mM tert-butyl hydroperoxide (TBH; Sigma-Aldrich) solution to achieve a final concentration of 1 mM. Two, three, and four h after the start of treatments, medium was removed, washed with 500 µL of PBS, and cells were harvested with 150 µL trypsin at 37 °C for 5 min. For each well, 1 mL of medium was added to stop the trypsin reaction, cells were centrifuged (149× *g*, 5 min), and the supernatant was discarded. A working solution with 5 µM in phenol red-free, FCS-free medium was prepared from the 40 mM 2′,7′-dichlorofluorescein diacetate (DCFH-DA; Sigma-Aldrich) stock solution, and the cell pellets were resuspended in 1 mL of this solution. After intracellular esterases increase the polarity of the molecule by deacetylation and prevent it from leaving the cell, the green fluorescent dichlorofluorescein (DCF) is formed by oxidation by various ROS [39]. After incubation at 37 °C for 30 min in the dark, the cells were centrifuged, the supernatant was discarded, and the cells were covered once again with PBS. Subsequently, pellets were resuspended in 400 µL of phenol red-free, FCS-free medium and were transferred to a flow cytometry tube. Flow cytometric analysis followed with absorbance at 488 nm and detection of emission at 530 nm (NovoCyte^®^ 2060R flow cytometer, Agilent Technologies Inc.). For this purpose, 10,000 events per sample were measured and the experiment was performed three times, independently.

### 2.7. In Vivo Mouse Experiment

Six-week-old female C57BL/6N^Crl^ mice were purchased from Charles River Laboratory (Wiga, Germany) and accommodated in the animal care institution at the University of Tuebingen. After one week, C57BL/6N^Crl^ females received a subcutaneous (s.c.) injection of 1 × 10^5^ B16F10 cells suspended in 50 μL sterile PBS (Sigma-Aldrich) into the right flank. Once tumors were palpable, the mice were randomly divided into four groups (eight mice as controls and six mice for each treatment) and respective treatments were started. A total of 42 mice were injected, but only mice that had uniform tumor size at treatment initiation were used for randomization. All treatments were applied intraperitoneally (i.p.). The anti-PD1 antibody J43 was injected at 2 mg/kg bodyweight in 100 μL isotonic saline solution, twice weekly. Ascorbate (Pascorbin^®^) was injected daily at 3 g/kg bodyweight. Mixed syringes were used for combined treatments (up to 700 μL volume for i.p. injections; 23G needle) to minimize pain in mice by reducing the number of i.p. injections. The same volume of solvent (NaCl (Sigma-Aldrich)) was used for up to 20 i.p. injections for the control group. Animals were weighed every other day through the experiment and tumor size was evaluated daily (volume = length × width × width × 0.5). To directly compare tumor volumes and weights between the treatment groups, all animals in the four treatment groups were sacrificed by using CO_2_ on the same day that sham-treated mice reached the abortion criterion. Tissue samples (organs, blood, and tumors) were collected and stored for further analysis. 

### 2.8. Immunohistochemistry of Murine Tumors and Organs 

Murine tumors were initially fixed in formalin (4%), embedded in paraffin, serially sectioned, and finally stained with hematoxylin and eosin (H&E). Proteins of interest were stained using antibodies against CD3 (Dianova, Hamburg, Germany; DIA-303), PCNA (Abcam; #ab92552), hypoxia-inducible factor 1α (HIF-1α; Biozol, Eching, Germany; #IHC-IW-PA 1041), and GLUT-1 (Abcam, Berlin, Germany; #ab115730). Subsequently, bound antibodies were stained with the ultraView Universal Alkaline Phosphatase Red Detection Kit from Ventana (Roche, Rotkreuz; Switzerland). Quantification of immunohistochemical staining was performed as follows. Five representative samples were stained per group (only four tumors could be evaluated for Glut-1 staining of the combination group). For the proliferation marker Pcna, the percentage of tumor cells with a positively stained nucleus was calculated using the formula [% positive cells] = (100 ∗ [number of positive tumor nuclei]/[total nuclei]), and the average value of three high magnifications per sample was calculated. For Glut-1, only the membranous signal was evaluated. Similar to the HercepTest^TM^, a score of 0–3 was defined for every sample, where 0 means that no staining or membrane staining is detected in <10% of B16F10 cells; 1 means that weak/weak perceptible membrane staining is detected in >10% of B16F10 cells; 2 means that weak-to-moderate complete membrane staining is detected in >10% of B16F10 cells; 3 means that strong complete membrane staining is detected in >10% of B16F10 cells. For CD3, the absolute number of CD3-positive cells in a representative non-necrotic tumor area of 1 mm^2^ per sample was determined.

### 2.9. Statistics

Statistics were calculated with GraphPad Prism version 8.4 (GraphPad Software, San Diego, CA, USA). One-way ANOVA and Tukey’s or Dunnet’s test for multiple comparisons were performed to calculate *p*-values and determine significances when comparing multiple groups. *p*-values ≤ 0.05 were assumed statistically significant (*: *p* ≤ 0.05; **: *p* ≤ 0.01; ***: *p* ≤ 0.001; ****: *p* ≤ 0.0001). For the quantification of the immunohistochemistry (IHC) staining the Kruskal–Wallis with Dunn’s post hoc test was used.

### 2.10. Ethics

Investigations were carried out in accordance with the Declaration of Helsinki [40]. The mice experiments were conducted in agreement with the European Union and German law and were approved by local authorities (Regierungspraesidium Tuebingen, HT3/16). 

## 3. Results

### 3.1. Growth-Inhibiting Effects of Ascorbate on B16F10 Cells

**Figure 1:**
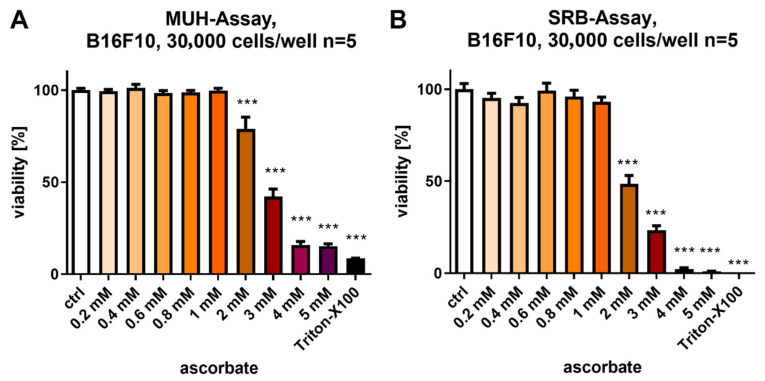
Ascorbate-induced growth inhibition on murine melanoma cells. (**A**) B16F10 mouse melanoma cells were exposed to varying amounts of ascorbate (0–5 mM). The viability of murine melanoma cells was evaluated using the MUH assay in comparison to the medium-treated control. Depicted are the mean values ± SD of five standalone experiments, measured in duplicate, respectively, and standardized to the medium-treated control. (**B**) B16F10 mouse melanoma cells were exposed to varying amounts of ascorbate (0–5 mM). The cell viability was assessed using the SRB assay in comparison to the medium-treated control. Depicted are the mean values ± SD of five standalone experiments, each measured in duplicate and standardized to the medium-treated control. ***: *p* ≤ 0.001. MUH, 4-methylumbelliferyl heptanoate; SD, standard deviation; SRB, sulforhodamine B.

To evaluate the direct effects of ascorbate, different concentrations of ascorbate were used to treat B16F10 cells (0–5 mM). Cell viability was determined using the fluorometric MUH assay after 24 h treatment with Triton-X100 as a positive control for complete cell death. Figure 1A shows that ascorbate concentrations of ≥2 mM reduced cell viability significantly, whereas ≥ 3 mM of ascorbate decreased the viability of B16F10 cells below a threshold of 50%, suggesting relevant antitumoral effects. These results were confirmed by the SRB assay, which determines the total protein content of the treated B16F10 cells and is proportionate to the number of living tumor cells (Figure 1B). Interestingly, the ascorbate effect was even more pronounced, decreasing the cell number below 50% with ≥2 mM of ascorbate.

**Figure 2:**
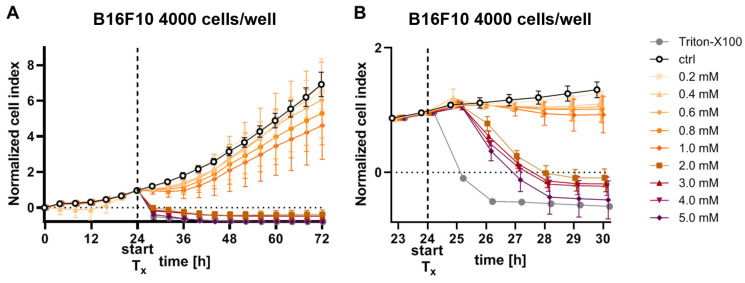
Real-time antiproliferative activity of ascorbate on B16F10 cells. B16F10 mouse melanoma cells were treated with varying amounts of ascorbate (0–5 mM). Cellular viability of treated wells was monitored by impedance measurement at 30 min intervals and compared to the medium-treated control. (**A**) The viability is shown in 4 h intervals over the complete period of 72 h. (**B**) The viability is shown in 1 h intervals in the first 6 h after treatment start. Depicted are the mean values ± SD of three stand-alone experiments, measured in quadruplicate, respectively (*n* = 11 for controls, *n* = 12 for treated cells). SD, standard deviation.

Based on the endpoint assays above, pharmacological ascorbate has a potent antitumor effect when appearing in the milieu of the melanoma cells. Although millimolar plasma levels of ascorbate are rapidly reached in the patient after infusion of ascorbate, they are also renally excreted within a short period of time. Accordingly, it is important to determine how long ascorbate must be in contact with B16F10 cells for to produce relevant antitumor effects. To determine the exact temporal onset and the extent of ascorbate effects in a real-time setting, B16F10 cells were treated with concentrations 0–5 mM and measured in real-time with the xCELLigence^®^ SP system (Agilent Technologies Inc.). Cell proliferation was tracked by measuring electrical impedance every 30 min over 72 h (Figure 2A). Detailed analysis revealed a fast antiproliferative effect of ascorbate treatment starting from 2 mM after 4 h of treatment (Figure 2B).

### 3.2. Effects of Ascorbate on ROS Production of Melanoma Cells 

**Figure 3:**
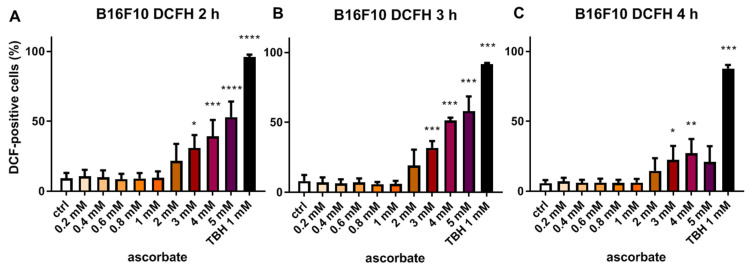
Effects of ascorbate on ROS production in melanoma cells. B16F10 cells (30,000 cells/well), were treated with ascorbate (0–5 mM), and measured flow-cytometrically 2 h (**A**), 3 h (**B**), and 4 h (**C**) after treatment. Shown are the mean values ± SD of DCF-positive cells of three independent experiments, indicating cells with elevated intracellular ROS levels. *: *p* ≤ 0.05; **: *p* ≤ 0.01; ***: *p* ≤ 0.001; ****: *p* ≤ 0.0001. DCF, 2′,7′-dichlorofluorescein (oxidized form); DCFH, 2′,7′-dichlorofluorescein (reduced form); ROS, reactive oxygen species; TBH, tert-butyl hydroperoxide; SD, standard deviation.

After having established that ascorbate in a concentration of ≥2 mM could quickly achieve antitumoral effects, we sought to determine the timing and extent of ascorbate-induced ROS formation and whether these actually correlate with cell death. Therefore, a DCFH-DA assay for ROS detection was performed (Figure 3). After treatment of B16F10 melanoma cells with ascorbate (0–5 mM), we observed that significant ROS production occurred with ascorbate levels ≥ 3 mM already after 2 h, with a peak at 3 h. These results were largely in line with the viability assays, which showed significant antitumoral effects with ≥ 2 mM ascorbate. 

### 3.3. Effects of Ascorbate and the Anti-PD1 Antibody J43 In Vivo

**Figure 4:**
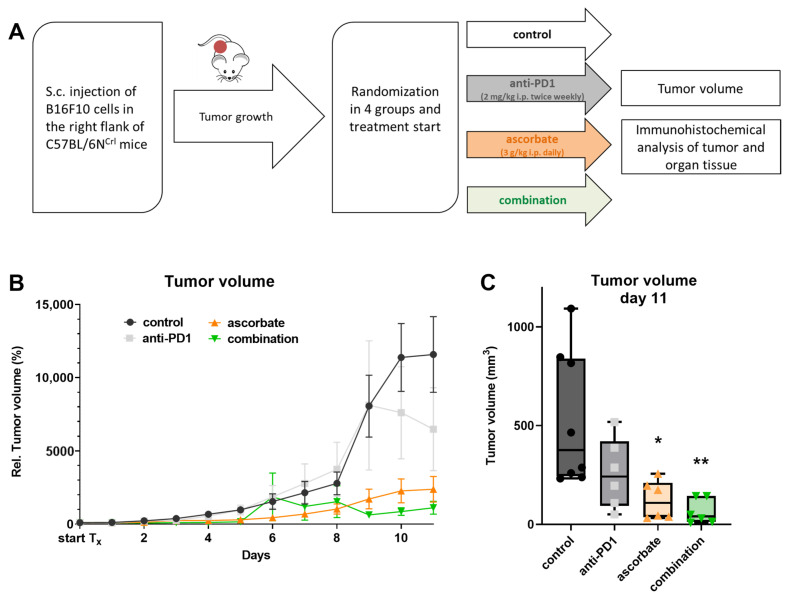
Effects of ascorbate and anti-PD1 in vivo in a B16F10 mouse model injected subcutaneously with the B16F10 melanoma cell line. (**A**) In vivo mouse experiment treatment regimen. (**B**) Relative tumor volume progression of female C57BL/6N^Crl^ during ascorbate treatment (3 g/kg i.p., daily), anti-PD1 treatment (2 mg/kg i.p., twice per week), and the combination of ascorbate and anti-PD1 treatment. (**C**) Endpoint measurement of tumor volume at day 11 after treatment initiation with ascorbate (3 g/kg i.p., daily), antiPD1 (2 mg/kg i.p., twice per week), and both treatments combined. Relative tumor volumes were calculated in relation to the tumor volume at treatment initiation, respectively. Mean values of normalized tumor volume ± SEM are shown. *: *p* ≤ 0.05; **: *p* ≤ 0.01. i.p., intraperitoneal; PD1, programmed cell death protein 1, SEM, standard error of the mean.

The evaluation of the effectiveness of treatment was carried out by s.c. injecting the syngeneic murine melanoma cell line B16F10 in the right flank of female C57BL/6N^Crl^ mice that were treated with ascorbate, anti-PD1 immunotherapy, or the combination of both in the following. This represents a fully immunocompetent murine melanoma model. Once the tumors had developed, female C57BL/6N^Crl^ mice were divided into four randomized groups (eight in the control group and six for each treatment), and treatment was started. Mice were treated via i.p. injections. Anti-PD1 antibody J43 was injected at 2 mg/kg body-weight, twice per week. Ascorbate was administered daily as injection at 3 g/kg bodyweight. In the combination treatment, both compounds were mixed immediately before application. The control group received the corresponding volume of isotonic saline as treatment. The treatment scheme is illustrated in Figure 4A. The weight of the mice was assessed every other day during the entire experiment. To determine the tumor volume, the length and width of each tumor were measured daily (volume = length × width × width × 0.5) (Figure 4B). To enable intertreatment group comparison, the mice of all four cohorts were sacrificed on day 11, 1 h after therapy initiation, with CO_2_, as soon as the animals in the control group reached the abortion criteria due to tumor size. 

All tumors of treated mice (either with ascorbate, anti-PD1, or the combination of ascorbate and anti-PD1) showed decreased tumor volumes compared to control. Tumors in the groups receiving either ascorbate or the combination treatment displayed a significant decrease in tumor volumes compared to control tumors at the end of the experiment (Figure 4C). To represent tumor growth over time, the calculated tumor volumes over time are relative to day 0 (therapy initiation) with 100% tumor volume (Figure 4B). Anti-PD1 treatment with J43 (grey) decreased the tumor growth compared to the sham-treated group (black). Similarly, high-dose ascorbate (orange) significantly reduced tumor growth. The combined treatment with ascorbate and anti-PD1 (green) led to less tumor growth in comparison to the control group and the groups treated with ascorbate or anti-PD1 alone. 

**Figure 5:**
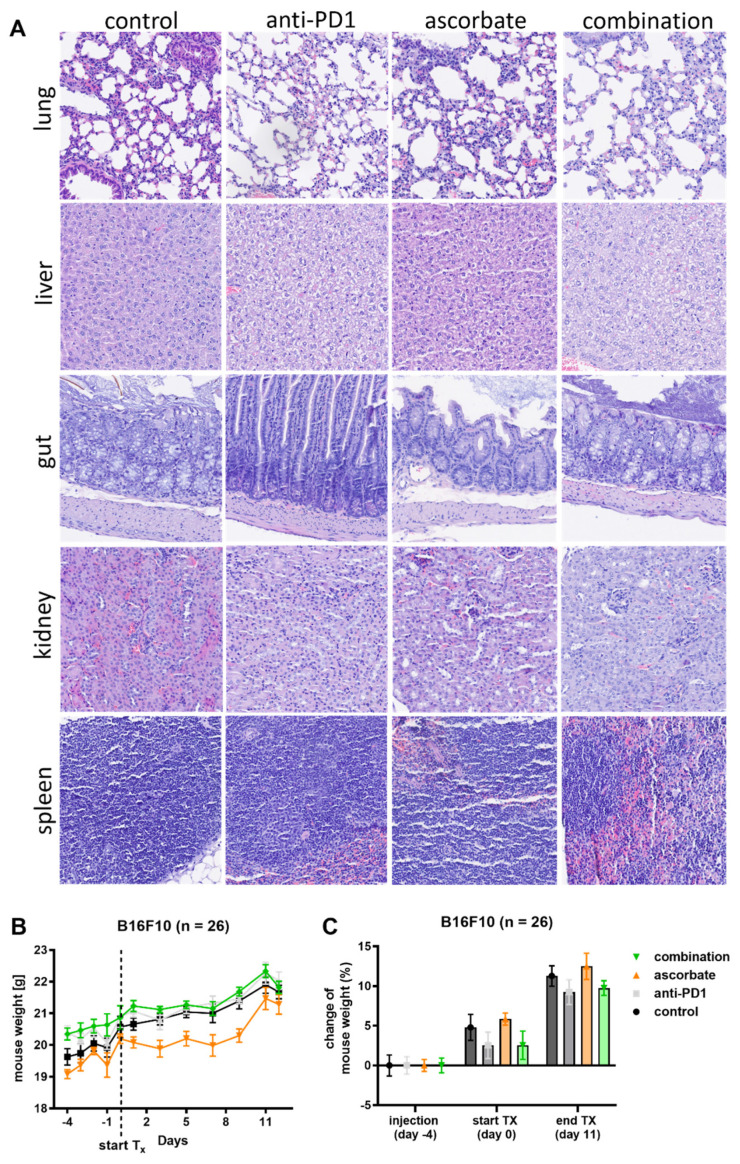

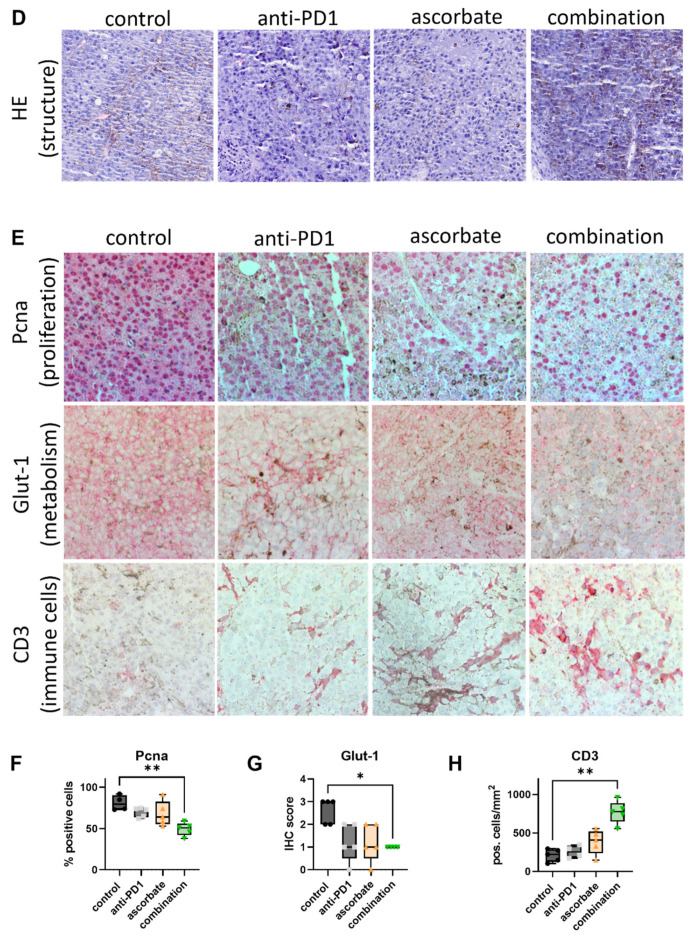
Effects of treatment with ascorbate and/or anti−PD1 on mouse organs and tumors. (**A**) H&E staining of lung, liver, gut, kidney, and spleen from animals after treatment either with ascorbate (3 g/kg i.p., daily), anti−PD1 (2 mg/kg i.p., twice per week), or the combination of ascorbate and anti−PD1. (**B**) Weight progression curves of animals treated with ascorbate (3 g/kg i.p., daily), anti−PD1 (2 mg/kg i.p., twice per week), or the combination of ascorbate and anti−PD1. (**C**) Percentage change in mouse weight during the experimental procedure. (**D**) H&E staining of the tumors of the groups treated with ascorbate (3 g/kg i.p., daily), anti−PD1 (2 mg/kg i.p., twice per week), or the combination of both. (**E**) Immunohistochemical tumor staining for Pcna, Glut−1, and CD3 after treatment with ascorbate (3 g/kg i.p., daily), anti−PD1 (2 mg/kg i.p., twice per week), or the combination of ascorbate and anti−PD1. Representative images are shown at 200× magnification. (**F**) Quantification of Pcna from tumors treated with ascorbate (3 g/kg i.p., daily), anti−PD1 (2 mg/kg i.p., twice per week), and the combination of both (*n* = 5 in every group). Displayed are the percentages of positive cells. (**G**) Quantification of Glut−1 from tumors treated with ascorbate (3 g/kg i.p., daily), anti−PD1 (2 mg/kg i.p., twice per week), and the combination of both (*n* = 5 in every group, except the combination which represents *n* = 4). Displayed is the IHC score. (**H**) Quantification of CD3-positive cells in tumors treated with ascorbate (3 g/kg i.p., daily), anti−PD1 (2 mg/kg i.p., twice weekly), and the combination of both (*n* = 5 in every group). Plotted is the number of CD3-stained cells per mm^2^. Treatment groups were statistically compared by using the Kruskal–Wallis test with Dunn’s post hoc analysis. Significant differences between groups are denoted with asterisks. *: *p* ≤ 0.05; **: *p* ≤ 0.01. Glut−1, glucose transporter 1; H&E, hematoxylin and eosin; IHC, immunohistochemistry; i.p., intraperitoneal; Pcna, proliferating cell nuclear antigen; PD1, programmed cell death protein 1.

To evaluate the state of health of the mice at the end of the experiment, various organs (lung, liver, intestine, kidney, and spleen) were examined histologically (Figure 5A). No changes in tissue microstructure or other cellular alterations were detected in the organs of the respective treatment groups. Moreover, animal weight revealed no significant differences between the groups throughout the experiment (Figure 5B). The change in mouse weight of the different treatment groups was normalized to the day of s.c. tumor cell injection and is shown in Figure 5C. All treatment groups gained weight, and none of the groups differed significantly from the others in this respect throughout the experiment. In addition, tumors of all treatment groups were stained with H&E (Figure 5D), as well as for Pcna to assess proliferation status, glucose / dehydroascorbate transporter Glut-1 expression, and CD3 to determine the amount of T-cell infiltration (Figure 5E). Pcna expression levels were higher in control tumors than in tumors of the three different treatment groups (Figure 5E), with the quantification shown in Figure 5F. The results indicate the highest proliferation of the tumor cells in the control group and the lowest in the combination group. Glut-1 tended to be reduced in all treatment groups, including combination therapy, compared to the control group (Figure 5E,G). CD3-positive cells were enhanced by trend in the areas of tumors treated with ascorbate or anti-PD1, whereas the combination resulted in a significant rise in these cells in line with increased immune cell infiltration into the tumor (Figure 5E,H). 

## 4. Discussion

In this study, we demonstrate that highly dosed ascorbate exhibits cytotoxic effects on B16F10 murine melanoma cells both in vitro and in an immunocompetent mouse melanoma model (C57BL/6N^Crl^). SRB assay results and impedance analysis (xCELLigence^®^ SP system) showed that ascorbate reduced the viability of cancerogenic B16F10 cells with high efficacy (≥2 mM). Using MUH assay, a peak effect was observed at a concentration ≥ 3 mM. These results were confirmed by our ROS analysis and are in line with the described ROS induction in the literature [23,41,42]. In recent studies, it was demonstrated that antitumoral ascorbate levels are only achieved by parenteral—and preferably intravenous—administration, and that ascorbate acts as a prodrug for the ascorbyl radical (Asc^●−^) and H_2_O_2_ in the blood and interstitium, respectively [43,44]. This is in contrast to the classification of ascorbate as a general antioxidative radical scavenger [23,24,42]. Nanoparticles have also been developed to optimize pharmacokinetics and tumor targeting to reduce the required high amount of ascorbate. Various formulations exist for these ascorbate nanodeliverers. It has been demonstrated that ascorbate administered in this manner can effectively induce cytotoxicity in cancerous cells in vitro and suppress tumor growth in mice, e.g., ascorbyl palmitate together with paclitaxel in a murine B16F10 model [45,46]. 

In recent years, various studies demonstrated an effect of ascorbate on varying melanoma cell lines, as well as cell lines from other tumor entities [41]. In A375.S2 melanoma cells, for example, ascorbate caused cell cycle arrest and apoptosis [47]. In B16F10 mouse melanoma cells, high-dose ascorbate led to the induction of caspase-8-independent apoptosis via decrease in transferrin receptor-dependent cellular iron uptake [48,49]. Proliferation of B16F10 melanoma cells was inhibited by ascorbate-dependent regulation of the p53-p21 pathway [50]. 

Remarkably, plasma levels of up to 20 mM that are well tolerated by healthy cells in vitro [42], can be achieved safely in human tumor patients by the application of intravenous ascorbate [22,51,52]. Since a subset of patients with melanoma strongly benefits from the use of immunotherapies it is important to find suitable add-on therapy options that either improve their efficacy or mitigate the sometimes therapy-limiting side effects. In addition, there is an continuous need for new treatment strategies in case of primary or secondary resistance to checkpoint inhibition, which is observed in many melanoma patients [16].

However, the performed in vitro assays were not appropriate to investigate the effects of high-dose ascorbate therapy on the immune system and thus not suitable to investigate possible additive, synergistic, or even antagonistic effects caused by the combination of ascorbate and PD1 blockade, which was the main aim of this study. To overcome such shortfall, we used immunocompetent C57BL/6N^Crl^ mice with a syngeneic background to the B16F10 cells studied in vitro. The B16F10 cells were administered to the mice by s.c. injection and the effect of both the respective monotherapy (PD1 antibody J43) and ascorbate (3 g/kg bodyweight) was compared with the combination of both. Repeated i.p. doses of 3–4 g/kg bodyweight were also used in other murine tumor models and additionally in a murine colitis model [37,53,54,55]. The role of ascorbate as a prodrug for H_2_O_2_ and the generation of the ascorbyl radical is well established in the literature, and future studies should quantify this whenever possible [42]. An additive effect of the combination therapy could at least be demonstrated, and at the same time, no significant additional toxicities appeared for the combination compared to the monotherapies, respectively. The suppression of human A375 melanoma cells by ascorbate treatment and caspase-3 and caspase-9 activation, as well as induction of the Bcl-2- and Bax-mediated mitochondrial pathway, was already described by Chen and coworkers [56]. Evidence that the combination of high-dose ascorbate with immune checkpoint inhibitors could be beneficial was provided by Luchtel and colleagues. They describe good effects of high-dose ascorbate in combination with anti-PD1 therapy in a lymphoma mouse model [35], whereas Magri et al. demonstrated that the tumor volume in colon, pancreatic, and breast cancers was significantly reduced by the combined administration of ascorbate with anti-PD1 or anti-CTLA4 in mice [36]. Recently, a study by Peng et al. described the combination of pharmacological ascorbate with PD-L1 blockade in a murine renal carcinoma model. The combination showed increased intratumoral CD4^+^ and CD8^+^ infiltration and elevated IFN-γ levels, as well as downstream signaling [57]. To the best of our knowledge, we have shown for the first time that the combination of ascorbate and PD1 inhibition with J43 is both highly effective and well tolerated in an immunocompetent melanoma mouse model resembling BRAF wild-type melanomas.

However, one limitation of the study was the short treatment time window until the discontinuation criterion was reached in the control animals, as subcutaneous B16F10 tumors grow rapidly once tumors are established [58]. Since all animals in this study were sacrificed at the same time point, some of the tumors in the therapy groups remained very small. Larger-sized preclinical studies with longer medication periods need to be performed to investigate these promising beneficial effects of high-dose ascorbate on melanoma growth and antitumoral immune response. It also remains open if the mechanistical aspects underlying the increased antitumor effects of the combination therapy are completely different and independent from each other or if high-dose ascorbate can enable an improved immune response via the immune checkpoint inhibitors, not only because of the prooxidative mechanisms, but also epigenetic modulation, ten eleven translocation 2 (TET2) enzyme activation (tet methylcytosine dioxygenase 2), and regulation of cytokine expression [57,59]. In a review by Bedhiafi et al., the role of high-dose ascorbate and its ability to modify the immune response against cancer is nicely summarized, and the authors describe how it may help to overcome the resistance mechanisms against immune checkpoint blockades [59].

An increased initial tumor cell death caused by high doses of ascorbate accompanied by increased antigen release could be also an additional mechanism. It seems plausible that dying tumor cells release more tumor-specific antigens, which in turn could further facilitate the immune response of the host under immunotherapy and fuel the cancer immunity cycle [60]. However, this hypothesis has to be proven more extensively in mice (preclinical immunocompetent melanoma models) and humans (clinical trials). Although no signs of toxicity in the C57BL/6N^Crl^ mice were observed, long-term treatment must be carefully monitored, and clinical studies are required to evaluate this promising combination and optimize the results of anti-PD1-based immunotherapy against malignant melanoma.

## 5. Conclusions

Our data support the benefits of this dual treatment of immunotherapy with highly dosed ascorbate to target melanoma cells. Nevertheless, clinical trials could further evaluate the effects of ROS-inducing and redox-active agents such as pharmacological high-dose ascorbate in the context of malignant melanoma, as well as the role of a stimulated immune system through immune checkpoint inhibition.

## Data Availability

The data presented in this study are available on request from the corresponding author.

## References

[1] Paluncic J., Kovacevic Z., Jansson P.J., Kalinowski D., Merlot A.M., Huang M.L.-H., Lok H.C., Sahni S., Lane D.J.R., Des Richardson R. (2016). Roads to melanoma: Key pathways and emerging players in melanoma progression and oncogenic signaling. Biochim. Biophys. Acta.

[2] Apalla Z., Lallas A., Sotiriou E., Lazaridou E., Ioannides D. (2017). Epidemiological trends in skin cancer. Dermatol. Pract. Concept..

[3] Tsai J., Lee J.T., Wang W., Zhang J., Cho H., Mamo S., Bremer R., Gillette S., Kong J., Haass N.K. (2008). Discovery of a selective inhibitor of oncogenic B-Raf kinase with potent antimelanoma activity. Proc. Natl. Acad. Sci. USA.

[4] Adams R., Coumbe J.E.M., Coumbe B.G.T., Thomas J., Willsmore Z., Dimitrievska M., Yasuzawa-Parker M., Hoyle M., Ingar S., Geh J.L.C. (2022). BRAF inhibitors and their immunological effects in malignant melanoma. Expert Rev. Clin. Immunol..

[5] Chapman P.B., Hauschild A., Robert C., Haanen J.B., Ascierto P., Larkin J., Dummer R., Garbe C., Testori A., Maio M. (2011). Improved survival with vemurafenib in melanoma with BRAF V600E mutation. N. Engl. J. Med..

[6] Wolchok J.D., Kluger H., Callahan M.K., Postow M.A., Rizvi N.A., Lesokhin A.M., Segal N.H., Ariyan C.E., Gordon R.-A., Reed K. (2013). Nivolumab plus ipilimumab in advanced melanoma. N. Engl. J. Med..

[7] Larkin J., Chiarion-Sileni V., Gonzalez R., Grob J.-J., Rutkowski P., Lao C.D., Cowey C.L., Schadendorf D., Wagstaff J., Dummer R. (2019). Five-Year Survival with Combined Nivolumab and Ipilimumab in Advanced Melanoma. N. Engl. J. Med..

[8] Robert C., Ribas A., Wolchok J.D., Hodi F.S., Hamid O., Kefford R., Weber J.S., Joshua A.M., Hwu W.-J., Gangadhar T.C. (2014). Anti-programmed-death-receptor-1 treatment with pembrolizumab in ipilimumab-refractory advanced melanoma: A randomised dose-comparison cohort of a phase 1 trial. Lancet.

[9] Amaral T., Kiecker F., Schaefer S., Stege H., Kaehler K., Terheyden P., Gesierich A., Gutzmer R., Haferkamp S., Uttikal J. (2020). Combined immunotherapy with nivolumab and ipilimumab with and without local therapy in patients with melanoma brain metastasis: A DeCOG* study in 380 patients. J. Immunother. Cancer.

[10] Li B., VanRoey M., Wang C., Chen T.T., Korman A., Jooss K. (2009). Anti-programmed death-1 synergizes with granulocyte macrophage colony-stimulating factor--secreting tumor cell immunotherapy providing therapeutic benefit to mice with established tumors. Clin. Cancer Res..

[11] Peng W., Liu C., Xu C., Lou Y., Chen J., Yang Y., Yagita H., Overwijk W.W., Lizée G., Radvanyi L. (2012). PD-1 blockade enhances T-cell migration to tumors by elevating IFN-γ inducible chemokines. Cancer Res..

[12] Mahoney K.M., Rennert P.D., Freeman G.J. (2015). Combination cancer immunotherapy and new immunomodulatory targets. Nat. Rev. Drug Discov..

[13] Villani A., Scalvenzi M., Fabbrocini G., Ocampo-Candiani J., Ocampo-Garza S.S. (2021). Looking into a Better Future: Novel Therapies for Metastatic Melanoma. Dermatol. Ther. (Heidelb).

[14] Betof Warner A., Palmer J.S., Shoushtari A.N., Goldman D.A., Panageas K.S., Hayes S.A., Bajwa R., Momtaz P., Callahan M.K., Wolchok J.D. (2020). Long-Term Outcomes and Responses to Retreatment in Patients With Melanoma Treated With PD-1 Blockade. J. Clin. Oncol..

[15] Topalian S.L., Hodi F.S., Brahmer J.R., Gettinger S.N., Smith D.C., McDermott D.F., Powderly J.D., Sosman J.A., Atkins M.B., Leming P.D. (2019). Five-Year Survival and Correlates Among Patients With Advanced Melanoma, Renal Cell Carcinoma, or Non-Small Cell Lung Cancer Treated With Nivolumab. JAMA Oncol..

[16] Amaral T., Seeber O., Mersi E., Sanchez S., Thomas I., Meiwes A., Forschner A., Leiter U., Eigentler T., Keim U. (2020). Primary Resistance to PD-1-Based Immunotherapy-A Study in 319 Patients with Stage IV Melanoma. Cancers.

[17] Kaesler S., Wölbing F., Kempf W.E., Skabytska Y., Köberle M., Volz T., Sinnberg T., Amaral T., Möckel S., Yazdi A. (2019). Targeting tumor-resident mast cells for effective anti-melanoma immune responses. JCI Insight.

[18] Hilke F.J., Sinnberg T., Gschwind A., Niessner H., Demidov G., Amaral T., Ossowski S., Bonzheim I., Röcken M., Riess O. (2020). Distinct Mutation Patterns Reveal Melanoma Subtypes and Influence Immunotherapy Response in Advanced Melanoma Patients. Cancers.

[19] Cameron E., Pauling L. (1976). Supplemental ascorbate in the supportive treatment of cancer: Prolongation of survival times in terminal human cancer. Proc. Natl. Acad. Sci. USA.

[20] Cameron E., Pauling L. (1978). Supplemental ascorbate in the supportive treatment of cancer: Reevaluation of prolongation of survival times in terminal human cancer. Proc. Natl. Acad. Sci. USA.

[21] Nauman G., Gray J.C., Parkinson R., Levine M., Paller C.J. (2018). Systematic Review of Intravenous Ascorbate in Cancer Clinical Trials. Antioxidants.

[22] Schoenfeld J.D., Sibenaller Z.A., Mapuskar K.A., Wagner B.A., Cramer-Morales K.L., Furqan M., Sandhu S., Carlisle T.L., Smith M.C., Abu Hejleh T. (2017). O_2_^·−^ and H_2_O_2_-Mediated Disruption of Fe Metabolism Causes the Differential Susceptibility of NSCLC and GBM Cancer Cells to Pharmacological Ascorbate. Cancer Cell.

[23] Chen Q., Espey M.G., Sun A.Y., Lee J.-H., Krishna M.C., Shacter E., Choyke P.L., Pooput C., Kirk K.L., Buettner G.R. (2007). Ascorbate in pharmacologic concentrations selectively generates ascorbate radical and hydrogen peroxide in extracellular fluid in vivo. Proc. Natl. Acad. Sci. USA.

[24] Chen Q., Espey M.G., Sun A.Y., Pooput C., Kirk K.L., Krishna M.C., Khosh D.B., Drisko J., Levine M. (2008). Pharmacologic doses of ascorbate act as a prooxidant and decrease growth of aggressive tumor xenografts in mice. Proc. Natl. Acad. Sci. USA.

[25] Creagan E.T., Moertel C.G., O’Fallon J.R., Schutt A.J., O’Connell M.J., Rubin J., Frytak S. (1979). Failure of high-dose vitamin C (ascorbic acid) therapy to benefit patients with advanced cancer. A controlled trial. N. Engl. J. Med..

[26] Moertel C.G., Fleming T.R., Creagan E.T., Rubin J., O’Connell M.J., Ames M.M. (1985). High-dose vitamin C versus placebo in the treatment of patients with advanced cancer who have had no prior chemotherapy. A randomized double-blind comparison. N. Engl. J. Med..

[27] Padayatty S.J., Sun H., Wang Y., Riordan H.D., Hewitt S.M., Katz A., Wesley R.A., Levine M. (2004). Vitamin C pharmacokinetics: Implications for oral and intravenous use. Ann. Intern. Med..

[28] Hoffer L.J., Levine M., Assouline S., Melnychuk D., Padayatty S.J., Rosadiuk K., Rousseau C., Robitaille L., Miller W.H. (2008). Phase I clinical trial of i.v. ascorbic acid in advanced malignancy. Ann. Oncol..

[29] Stephenson C.M., Levin R.D., Spector T., Lis C.G. (2013). Phase I clinical trial to evaluate the safety, tolerability, and pharmacokinetics of high-dose intravenous ascorbic acid in patients with advanced cancer. Cancer Chemother. Pharmacol..

[30] Lv H., Wang C., Fang T., Li T., Lv G., Han Q., Yang W., Wang H. (2018). Vitamin C preferentially kills cancer stem cells in hepatocellular carcinoma via SVCT-2. NPJ Precis. Oncol..

[31] Ma Y., Chapman J., Levine M., Polireddy K., Drisko J., Chen Q. (2014). High-dose parenteral ascorbate enhanced chemosensitivity of ovarian cancer and reduced toxicity of chemotherapy. Sci. Transl. Med..

[32] Yun J., Mullarky E., Lu C., Bosch K.N., Kavalier A., Rivera K., Roper J., Chio I.I.C., Giannopoulou E.G., Rago C. (2015). Vitamin C selectively kills KRAS and BRAF mutant colorectal cancer cells by targeting GAPDH. Science.

[33] Ang A., Pullar J.M., Currie M.J., Vissers M.C.M. (2018). Vitamin C and immune cell function in inflammation and cancer. Biochem. Soc. Trans..

[34] Jeong Y.-J., Kim J.-H., Hong J.-M., Kang J.S., Kim H.-R., Lee W.J., Hwang Y. (2014). Vitamin C treatment of mouse bone marrow-derived dendritic cells enhanced CD8(+) memory T cell production capacity of these cells in vivo. Immunobiology.

[35] Luchtel R.A., Bhagat T., Pradhan K., Jacobs W.R., Levine M., Verma A., Shenoy N. (2020). High-dose ascorbic acid synergizes with anti-PD1 in a lymphoma mouse model. Proc. Natl. Acad. Sci. USA.

[36] Magrì A., Germano G., Lorenzato A., Lamba S., Chilà R., Montone M., Amodio V., Ceruti T., Sassi F., Arena S. (2020). High-dose vitamin C enhances cancer immunotherapy. Sci. Transl. Med..

[37] Niessner H., Burkard M., Leischner C., Renner O., Plöger S., Meraz-Torres F., Böcker M., Hirn C., Lauer U.M., Venturelli S. (2022). Therapeutic Efficacy of Pharmacological Ascorbate on Braf Inhibitor Resistant Melanoma Cells In Vitro and In Vivo. Cells.

[38] Schleich T., Rodemeister S., Venturelli S., Sinnberg T., Garbe C., Busch C. (2015). Decreased Plasma Ascorbate Levels in Stage IV Melanoma Patients. Metab. Nutr. Oncol..

[39] Wang X., Roper M.G. (2014). Measurement of DCF fluorescence as a measure of reactive oxygen species in murine islets of Langerhans. Anal. Methods Adv. Methods Appl..

[40] (2013). World Medical Association Declaration of Helsinki: Ethical principles for medical research involving human subjects. JAMA.

[41] Sinnberg T., Noor S., Venturelli S., Berger A., Schuler P., Garbe C., Busch C. (2014). The ROS-induced cytotoxicity of ascorbate is attenuated by hypoxia and HIF-1alpha in the NCI60 cancer cell lines. J. Cell. Mol. Med..

[42] Chen Q., Espey M.G., Krishna M.C., Mitchell J.B., Corpe C.P., Buettner G.R., Shacter E., Levine M. (2005). Pharmacologic ascorbic acid concentrations selectively kill cancer cells: Action as a pro-drug to deliver hydrogen peroxide to tissues. Proc. Natl. Acad. Sci. USA.

[43] Pei Z., Wu K., Li Z., Li C., Zeng L., Li F., Pei N., Liu H., Zhang S.-L., Song Y.-Z. (2019). Pharmacologic ascorbate as a pro-drug for hydrogen peroxide release to kill mycobacteria. Biomed. Pharmacother..

[44] Venturelli S., Leischner C., Helling T., Burkard M., Marongiu L. (2021). Vitamins as Possible Cancer Biomarkers: Significance and Limitations. Nutrients.

[45] Zhou M., Li X., Li Y., Yao Q., Ming Y., Li Z., Lu L., Shi S. (2017). Ascorbyl palmitate-incorporated paclitaxel-loaded composite nanoparticles for synergistic anti-tumoral therapy. Drug Deliv..

[46] Sun Y., Wang Z., Zhang P., Wang J., Chen Y., Yin C., Wang W., Fan C., Sun D. (2020). Mesoporous silica integrated with Fe3O4 and palmitoyl ascorbate as a new nano-Fenton reactor for amplified tumor oxidation therapy. Biomater. Sci..

[47] Lin S.-Y., Lai W.-W., Chou C.-C., Kuo H.-M., Li T.-M., Chung J.-G., Yang J.-H. (2006). Sodium ascorbate inhibits growth via the induction of cell cycle arrest and apoptosis in human malignant melanoma A375.S2 cells. Melanoma Res..

[48] Kang J.S., Cho D., Kim Y.-I., Hahm E., Kim Y.S., Jin S.N., Kim H.N., Kim D., Hur D., Park H. (2005). Sodium ascorbate (vitamin C) induces apoptosis in melanoma cells via the down-regulation of transferrin receptor dependent iron uptake. J. Cell. Physiol..

[49] Kang J.S., Cho D., Kim Y.-I., Hahm E., Yang Y., Kim D., Hur D., Park H., Bang S., Hwang Y.I. (2003). L-ascorbic acid (vitamin C) induces the apoptosis of B16 murine melanoma cells via a caspase-8-independent pathway. Cancer Immunol. Immunother..

[50] Hahm E., Jin D.-H., Kang J.S., Kim Y.-I., Hong S.-W., Lee S.K., Kim H.N., Da Jung J., Kim J.E., Shin D.H. (2007). The molecular mechanisms of vitamin C on cell cycle regulation in B16F10 murine melanoma. J. Cell. Biochem..

[51] Allen B.G., Bodeker K.L., Smith M.C., Monga V., Sandhu S., Hohl R., Carlisle T., Brown H., Hollenbeck N., Vollstedt S. (2019). First-in-Human Phase I Clinical Trial of Pharmacologic Ascorbate Combined with Radiation and Temozolomide for Newly Diagnosed Glioblastoma. Clin. Cancer Res..

[52] Renner O., Burkard M., Michels H., Vollbracht C., Sinnberg T., Venturelli S. (2021). Parenteral high-dose ascorbate—A possible approach for the treatment of glioblastoma (Review). Int. J. Oncol..

[53] Chen P., Stone J., Sullivan G., Drisko J.A., Chen Q. (2011). Anti-cancer effect of pharmacologic ascorbate and its interaction with supplementary parenteral glutathione in preclinical cancer models. Free Radic. Biol. Med..

[54] Zhang X., Liu T., Li Z., Feng Y., Corpe C., Liu S., Zhang J., He X., Liu F., Xu L. (2019). Hepatomas are exquisitely sensitive to pharmacologic ascorbate (P-AscH-). Theranostics.

[55] Kondo K., Hiramoto K., Yamate Y., Goto K., Sekijima H., Ooi K. (2019). Ameliorative Effect of High-Dose Vitamin C Administration on Dextran Sulfate Sodium-Induced Colitis Mouse Model. Biol. Pharm. Bull..

[56] Chen X.-Y., Chen Y., Qu C.-J., Pan Z.-H., Qin Y., Zhang X., Liu W.-J., Li D.-F., Zheng Q. (2019). Vitamin C induces human melanoma A375 cell apoptosis via Bax- and Bcl-2-mediated mitochondrial pathways. Oncol. Lett..

[57] Peng D., He A., He S., Ge G., Wang S., Ci W., Li X., Xia D., Zhou L. (2022). Ascorbic acid induced TET2 enzyme activation enhances cancer immunotherapy efficacy in renal cell carcinoma. Int. J. Biol. Sci..

[58] Serrano O.K., Parrow N.L., Violet P.-C., Yang J., Zornjak J., Basseville A., Levine M. (2015). Antitumor effect of pharmacologic ascorbate in the B16 murine melanoma model. Free Radic. Biol. Med..

[59] Bedhiafi T., Inchakalody V.P., Fernandes Q., Mestiri S., Billa N., Uddin S., Merhi M., Dermime S. (2022). The potential role of vitamin C in empowering cancer immunotherapy. Biomed. Pharmacother..

[60] Chen D.S., Mellman I. (2013). Oncology meets immunology: The cancer-immunity cycle. Immunity.

